# Effect of 2002 FIFA World Cup: Point of Attachment That Promotes Mass Football Participation

**DOI:** 10.3389/fpsyg.2022.857323

**Published:** 2022-03-30

**Authors:** Taeahn Kang, Jeongbeom Hahm, Hirotaka Matsuoka

**Affiliations:** ^1^Waseda Institute for Sport Sciences, Waseda University, Tokyo, Japan; ^2^Faculty of Sport Sciences, Waseda University, Tokyo, Japan

**Keywords:** FIFA world cup, mass football participation, points of attachment, identity theory, hierarchical regression analysis

## Abstract

The 2002 FIFA World Cup Korea/Japan significantly promoted football in the host countries. However, it remains unclear how the event has changed mass football (soccer in North America) participation. This study applies points of attachment (POA)—a well-developed concept in the field of sport management—to the 2002 FIFA World Cup and aims to examine which specific POA promoted football participation frequency immediately after the event and the present frequency of football participation in the host countries. An online questionnaire survey was conducted in South Korea (*n* = 405) and Japan (*n* = 398). The samples included adults aged > 19 as of the hosting date of the 2002 World Cup. Hierarchical regression analyses were performed to test all the datasets by employing four POAs (players, coaches, national teams, and football) as independent variables. Multiple control variables (e.g., nationality and age) and two dependent variables (football participation frequency immediately after the event and the present frequency of football participation) were included in the model. Correspondingly, those who had a higher attachment to each point during the event showed a higher frequency of football participation immediately after the event. In contrast, only two POAs (players and coaches) led to a higher frequency of present football participation. These findings provide the first empirical evidence highlighting the influence of the 2002 FIFA World Cup on mass football participation depending on the POA.

## Introduction

The 2002 FIFA World Cup Korea/Japan had a significant impact on the promotion of football—soccer in North America—in the host countries (Horne and Manzenreiter, 2004, 2013). Asia hosted the event for the first time, and the historic occasion has considerably changed the situation surrounding football (Manzenreiter and Horne, 2004; Kim and Petrick, 2005). For example, most of the stadiums used for the 2002 FIFA World Cup were newly built (17 of the 20) (Alm, 2012). These stadiums contributed to the infrastructure for professional or national football games after the event. The number of public football facilities has been increasing since 1996, the official announcement of the hosting decision: from 200 in 1996 (Ministry of Culture and Sports, 1996) to 1,185 in 2016 in South Korea (Ministry of Culture Sports and Tourism, 2016a), and from 2,331 in 1996 to 2,600 in 2018 in Japan (Portal Site of Official Statistics of Japan, 2020). It also led to the growth of professional football leagues, given the expansion in the number of clubs between 1996 and 2020: 13 clubs in South Korea (from 9 to 22) and 40 clubs in Japan (from 16 to 56). In addition, the number of student athletes in football in South Korea doubled to 26,812 in 2016 (Ministry of Culture Sports and Tourism, 2016a) compared to 10,692 in 1996 (Ministry of Culture and Sports, 1996), while there was a massive increase in spectators of Japanese Professional Football League Division 1 over the past two decades: from 13,353 per game in 1996 to 20,751 in 2019 (J.LEAGUE Data Site, 2020).

Despite these developments, it remains unclear how mass football participation has changed over time. Mass football participation refers to grassroots football participation, or football for all (Veal et al., 2012). Based on the trickle-down effect (Weed, 2009), mega-sporting events are believed to increase mass sport participation in host regions (Ramchandani et al., 2015; Weed et al., 2015; Potwarka and Leatherdale, 2016). However, there is limited research on such an impact in the context of the 2002 FIFA World Cup. National surveys conducted in each host country reported that football participation rates increased from 5.1% in 1994 (Ministry of Culture and Sports, 1994) to 9.1% in 2016 in South Korea (Ministry of Culture Sports and Tourism, 2016b) and from 2.3% in 1996 (Sasakawa Sports Foundation, 1996) to 4.7% in 2018 in Japan (Sasakawa Sports Foundation, 2018). However, such a growth seemed much lower compared to other developments (e.g., the infrastructure development for football, the growth of professional football leagues, and the increasing number of student athletes in football), implying that its effect on football participation was limited in host countries. Moreover, the 2002 FIFA World Cup was the first to be hosted by more than one nation, signifying the uniqueness or importance of this tournament compared to other World Cup events. Therefore, considering the importance of the trickle-down effect within a mega-sporting event (Hahm et al., 2020; Potwarka and Wicker, 2021), further studies are needed to identify the factors triggering the trickle-down effect in the context of the 2002 FIFA World Cup.

Accordingly, this study focuses on the points of attachment (POA) to the 2002 FIFA World Cup. POA refers to a psychological association with a specific entity (Kwon and Armstrong, 2004). Sports consumers usually have multiple points of attachment to a sports team or event (e.g., the team, player, coach, and sport; Trail et al., 2003; Kwon et al., 2005; Robinson and Trail, 2005). POA is considered an important source of consumption preference (Robinson et al., 2004; Hallmann et al., 2018). Based on the identity theory (Stryker, 1968, 1980), individuals who perceive their roles as fans of a sports property as meaningful for their life (i.e., those with a higher attachment) are more likely to be affected by the property (Kwon et al., 2005; Trail et al., 2005; Shapiro et al., 2013). This implies that the trickle-down effect of the 2002 FIFA World Cup on football participation could assume significance among those with high attachment to the event. Given that POA is a well-developed concept for predicting spectator behavior in the field of sport management (Funk et al., 2001; Mahony et al., 2002; Trail et al., 2003; Ballouli et al., 2016), an understanding of which POA predicts mass football participation can be beneficial for underlying the impact of the 2002 FIFA World Cup because of its potential role in weakening the controversy regarding the validity of the trickle-down effect that has been raised in previous studies (Tomlinson, 2014; Grix et al., 2017; Taks et al., 2018). Therefore, this study examines how attachment to each object related to the 2002 FIFA World Cup promoted football participation frequency immediately after the event and the present frequency of football participation in host countries.

The current study conducted an online questionnaire survey in South Korea and Japan. Hierarchical regression analyses were used to test the datasets by employing POA as independent variables. Several control variables (e.g., nationality and age) and two dependent variables (i.e., the football participation frequency immediately after the 2002 FIFA World Cup and the present frequency of football participation) were included in the regression models. The findings of this study contribute to the trickle-down effect of a mega-sporting event by providing an understanding of the impact of the 2002 FIFA World Cup on mass football participation, depending on POA.

## Theoretical Background and Hypothesis Development

### Trickle-Down Effect of Mega-Sporting Events on Sport Participation

The trickle-down effect best illustrates how mega-sporting events promote mass sport participation in host communities (Weed, 2009; Ramchandani et al., 2015; Weed et al., 2015; Potwarka and Leatherdale, 2016). The trickle-down effect refers to “a process by which people are inspired by the elite sport, sports people, or sports events to participate themselves” (Weed, 2009, p. 4). Its framework is based on the economic literature that analyzes economic and social issues, such as inequalities of growth and wealth in capital markets. As wealth trickles from the top (i.e., the haves) to the bottom (i.e., the have nots), either driven by investments or a state policy of redistribution, the economy gives rise to more significant opportunities for all (Aghion and Bolton, 1997). The trickle-down effect has been widely applied in the context of mega-sporting events (Potwarka and Wicker, 2021). Weed et al. (2015) stated that sport participation is internally motivated by external forces, such as elite sports, athletes, and events. Extensive studies in sport management have highlighted the critical role of mega-sporting events or sporting success in enhancing mass sport participation (Veal et al., 2012; Ramchandani et al., 2015; Chen and Henry, 2016; Potwarka and Leatherdale, 2016; Potwarka et al., 2018; Kokolakakis et al., 2019; Cleland et al., 2020; Hahm et al., 2020).

Although the positive outcomes of the trickle-down effect in the sporting context have been demonstrated, controversy regarding its validity has also been highlighted in recent research. Empirical evidence on the weaker links between mega-sporting events and mass sport participation in host regions than what was promised by event organizers or policymakers has been observed by Tomlinson (2014), Stewart and Rayner (2016), and Grix et al. (2017). The evidence contradicts the effectiveness of the trickle-down effect and leads to the conclusion that it is political rhetoric as a means of securing hosting rights to offset public spending (Charlton, 2010; Sousa-Mast et al., 2013; Chalip et al., 2017; Taks et al., 2018). This debate on the trickle-down effect has raised the necessity of further studies that explore “when,” “where,” and “how” individuals in the host regions are influenced by mega-sporting events (Veal et al., 2012; Hahm et al., 2020; Potwarka and Wicker, 2021). Particularly, given the results of Weed et al.’s (2015) systematic review highlighting that the potential of mega-sporting events for boosting mass sport participation in host countries can only be leveraged by additional investments and relevant actions, the delivery process of the trickle-down effect cannot be realized without specific designs for the participation legacy. Therefore, more studies identifying the conditions under which the trickle-down effect of mega-sporting events on mass sport participation has remained should be conducted to better understand its impact (Hahm et al., 2020; Potwarka and Wicker, 2021).

### Points of Attachment

Points of attachment (POA) is the most frequently investigated theoretical concept for understanding sports consumer behavior (Trail et al., 2003; Robinson et al., 2004; Shapiro et al., 2013; Pan and Phua, 2020). POA is defined as a psychological connection to a specific entity (Kwon and Armstrong, 2004). It consists of multidimensional constructs within the context of sports settings such as attachment to sports, teams, players, and coaches (Trail et al., 2003; Kwon et al., 2005; Robinson and Trail, 2005). Multidimensionality has been acknowledged in developing POA scales and has been utilized in various sports settings (Wann and Branscombe, 1993; Trail et al., 2003; Hallmann et al., 2018). Previous studies have demonstrated multiple ways in which sports fans exhibit their attachment by highlighting that those with different POAs show diverse sports-related behaviors (Trail et al., 2003; Robinson et al., 2004; Spinda et al., 2016).

Identity theory provides a framework for understanding POA, which is interchangeably used with role identification—“a set of meanings applied to the self in a social role or situation, defining what it means to be who one is in that role or situation” (Burke and Stets, 1999, p. 349) (Laverie and Arnett, 2000; Kwon et al., 2005; Trail et al., 2005; Shapiro et al., 2013). Identity theory has been developed with the key proposition that people’s role identities are guided by past behaviors and predict future behaviors (Stryker, 1968, 1980). Identity is defined as “parts of a self, composed of the meanings that persons attach to the multiple roles they typically play in highly differentiated contemporary societies” (Stryker and Burke, 2000, p. 284). This role signifies an identity standard, which is a cognitive representation of what a particular role entails (Stryker and Burke, 2000). A specific identity is a set of beliefs regarding the importance of an individual’s role (Ervin and Stryker, 2001). Role salience is a central concept in identity theory owing to its part in creating identity standards (Lock and Heere, 2017). Several factors can affect sports fans’ identity salience, including situational involvement, enduring involvement, and attachment (Laverie and Arnett, 2000). Different roles that an individual occupies are usually of variable importance (Callero, 1985). Considering these multidimensional aspects, the importance of playing a single role refers to the enduring placement of role identity in one’s self-concept (Lock and Funk, 2016). For example, an individual who perceives being a sports fan to be more important is more likely to be a sports fan. Contrarily, if a sports fan is less important than his/her other roles in the workplace or elsewhere, he/she is less likely to behave like a sports fan. Therefore, identity salience can positively impact sporting event attendance (Laverie and Arnett, 2000) and cognitive and affective responses to team-related stimuli (Trail et al., 2017). Furthermore, identity standards generally interact with perceived situational meanings and cause a cognitive comparison between ideal and situationally impacted roles (Stryker and Burke, 2000). The importance of having a single role is related to the social positions that an individual occupies (Callero, 1985). Accordingly, identity theory is considered essential for understanding POA (or role identification) (Lock and Heere, 2017; Pan and Phua, 2020).

From the viewpoint of identity theory, various roles are often formed among sports consumers based on POA (Woo et al., 2009; Ballouli et al., 2016). Initial studies on POA identified a seven-factor model: attachment to the player, team, coach, community, type of sports, university, and level of sports (Trail and James, 2001; Trail et al., 2003). Essentially, sports fans may play seven different roles in their minds. In addition, such a POA model has varied through extensive research, and POA dimensions are usually determined by the research context (Kwon and Armstrong, 2004; Robinson et al., 2004; Kwon et al., 2005; Woo et al., 2009; Shapiro et al., 2013; Ballouli et al., 2016; Spinda et al., 2016; Hallmann et al., 2018). This indicates the importance of contemplating the research context when developing POA dimensions. Based on these arguments, this study employs four dimensions of POA, carefully considering the research context: attachments to the player, coach, national team, and football itself. The FIFA World Cup is a top-level football tournament worldwide. Attachment to the level of sports is often used in relatively low-level sporting events compared to competitions like the FIFA World Cup (e.g., Trail et al., 2003; Kwon et al., 2005; Woo et al., 2009). Therefore, the competition level dimension was not employed in this study. Moreover, three POAs—toward the team, community, and university—likely share a similar point of “the national team” in the present study. A single dimension of POA, attachment to the community, is thereby applied to the research with the specification of the POA name to “the attachment to the national team.” Eventually, attachment to players, coaches, national teams, and football was employed in the current study.

### Points of Attachment to the 2002 FIFA World Cup and Football Participation

A high degree of points of attachment (POA) can be a critical condition under which the trickle-down effect of the 2002 FIFA World Cup on football participation has arisen among residents of host countries. According to the identity theory (Stryker, 1968, 1980) and role salience (Lock and Heere, 2017), for individuals who have a high attachment to the 2002 FIFA World Cup during the event, their role as fans is likely to become meaningful in their lifetime. Therefore, they are expected to be affected by the trickle-down effect of the 2002 FIFA World Cup more than those with a low attachment, which possibly brings about a higher frequency of football participation immediately after the 2002 FIFA World Cup. Additionally, drawing on the aspect of participation “legacy” of mega-sporting events (Weed et al., 2015; Hahm et al., 2020), individuals with high attachment are likely to have a high frequency of football participation to date. Conversely, for those with low attachment, the role of being a fan of the 2002 FIFA World Cup can be considered less important. Therefore, the relatively low trickle-down effect of the 2002 FIFA World Cup is expected to occur in football participation. Based on the above arguments, the following hypotheses were proposed:

*Hypothesis 1a: The higher attachment to the player during the 2002 FIFA World Cup positively impacts football participation frequency immediately after the event.*
*Hypothesis 1b: The higher attachment to the player during the 2002 FIFA World Cup positively impacts the present frequency of football participation.*
*Hypothesis 2a: The higher attachment to the coach during the 2002 FIFA World Cup positively impacts football participation frequency immediately after the event.*
*Hypothesis 2b: The higher attachment to the coach during the 2002 FIFA World Cup positively impacts the present frequency of football participation.*
*Hypothesis 3a: The higher attachment to the national team during the 2002 FIFA World Cup positively impacts football participation frequency immediately after the event.*
*Hypothesis 3b: The higher attachment to the national team during the 2002 FIFA World Cup positively impacts the present frequency of football participation.*
*Hypothesis 4a: The higher attachment to football during the 2002 FIFA World Cup positively impacts football participation frequency immediately after the event.*
*Hypothesis 4b: The higher attachment to football during the 2002 FIFA World Cup positively impacts the present frequency of football participation.*

### Control Variables

This research controls for several demographics and experiences with the 2002 FIFA World Cup for more accurate testing of the hypotheses. The 2002 FIFA World Cup was co-hosted by two countries. According to Yi and Park (2003), Kim et al. (2009), and Byon et al. (2014), diverse cultural backgrounds generate different attitudes and behaviors. Therefore, the nationality of an individual is controlled in this study. Age was also employed as a control variable since it has been used in various research fields (e.g., Cunningham, 2021; Hahm et al., 2021; Kang and Matsuoka, 2021). As for experiences regarding the 2002 FIFA World Cup, four variables were applied to this study. Studies on the FIFA World Cup state that spectatorship patterns (i.e., active vs. passive) and the degree of spectator experience during the event could lead to different consumer behavior outcomes (Hahm et al., 2020, 2021). Thus, an individual’s experiences watching a game of the 2002 FIFA World Cup at the stadium, on TV, and at the public viewing site were included in the control variables. Finally, inspiration from experience with the 2002 FIFA World Cup was controlled. Inspiration is defined as a positive motivational state triggered by external environments such as someone or something. It targets specific behaviors (Thrash and Elliot, 2003). According to Hahm et al. (2020), inspiration from experience with a mega-sporting event can significantly enhance mass sport participation in the host region. However, considering the research purpose of this study, which aims to examine the effects of four POAs on football participation frequencies, the authors employ inspiration from experience with the 2002 FIFA World Cup as a control variable.

## Materials and Methods

### Data Collection and Participants

An online questionnaire survey was administered to participants from large online panel recruitment companies in South Korea and Japan between October and November 2019. The participants were adult males aged over 19, as of the hosting date of the 2002 FIFA World Cup. They were above 36 years of age at the time of the survey. Those under 19 years of age were in different environments—educational institutions—during the 2002 FIFA World Cup, which might have led to varied reactions from adult participants. This study also investigated whether the frequency of football participation increased after the 2002 FIFA World Cup. This signifies that those who have played football at least once a year before the 2002 FIFA World Cup host are eligible for this research. However, the authors faced difficulty recruiting female participants since very few had such experience playing football at that time. Based on the survey of the Sasakawa Sports Foundation (1996), only a single Japanese woman had experience in 1996 among 809 women (0.1%); a survey by the Ministry of Culture and Sports (1994) found that only 0.2% had experience in 1994 among 1,313 South Korean women. However, although there was a marginal increase to 1.7% among Japanese women in 2018 (Sasakawa Sports Foundation, 2018) and to 1.1% among South Korean women in 2016 (Ministry of Culture Sports and Tourism, 2016b), it could not address the challenge of recruiting female samples. Therefore, after cross-checking the conditions of the survey subjects required for the study with football participation in both countries, all eligible participants were males.

Prior to the survey, the authors prepared three questions to test participants’ recall of the 2002 FIFA World Cup: the names of the host countries, the winner, and the final result achieved by the national team. A total of 803 samples, 405 from South Korea and 398 from Japan, who provided correct answers to the questions, were finally included in the main survey. The average age of South Korean sample was 44.2 years, ranging from age 36–58, and the average age of Japanese samples was 47.4 years, ranging from age 37–65. Considering the nationwide characteristics of the FIFA World Cup, they were recruited without being geographically biased. At the initial stage of the main survey, they were given a short scenario from the 2002 FIFA World Cup. It was created to help them recall their memories. After reading it, they answered questions about their experiences with the 2002 FIFA World Cup, inspiration from the experience with the 2002 FIFA World Cup (i.e., control variables), four POAs to the 2002 FIFA World Cup (i.e., independent variables), and two football participation frequencies (i.e., the football participation frequency immediately after the 2002 FIFA World Cup and the present frequency of football participation; dependent variables).

### Measurements and Data Analyses

The items considered the four POAs to the 2002 FIFA World Cup were adapted from Trail et al. (2003): attachment to the player with three items, attachment to the coach with three items, attachment to the national team with three items, and attachment to football with three items (Table 1). These items were rated on a 7-point Likert-type scale ranging from 1 = *strongly disagree* to 7 = *strongly agree*. One item rated on a 7-point Likert-type scale was adopted from Shamir and Ruskin (1984) to measure football participation frequency immediately after the 2002 FIFA World Cup (i.e., *immediately after the 2002 FIFA World Cup, I played football more frequently than I normally did before the 2002 FIFA World Cup*) and the present frequency of football participation (i.e., *of late, I am playing football more frequently than I normally did before the 2002 FIFA World Cup*). These items were measured based on the total number of times an individual played football. Lastly, inspiration from the experience of the 2002 FIFA World Cup was assessed using three items (Thrash and Elliot, 2003), rated on a 7-point Likert-type scale.

**TABLE 1:** Measurement scales and results of confirmatory factor analysis.

Construct items	*M*	*SD*	λ	CR	AVE
**Inspiration from experience with the 2002 FIFA World Cup**				0.92	0.80
I experienced inspiration when I experienced the 2002 FIFA World Cup.	6.21	1.07	0.92		
Something I experienced inspired me when I experienced the 2002 FIFA World Cup.	6.08	1.12	0.88		
I felt inspired frequently when I experienced the 2002 FIFA World Cup.	6.22	1.00	0.88		
**Attachment to the player during the 2002 FIFA World Cup**				0.88	0.72
I identified with the individual player on the team more than with the team.	4.47	1.64	0.78		
I was a big fan of a specific player more than I was a fan of the team.	4.30	1.71	0.85		
I considered myself a fan of a certain player rather than a fan of the team.	4.28	1.72	0.90		
**Attachment to the coach during the 2002 FIFA World Cup**				0.91	0.77
I am a big fan of (coach name).	4.77	1.70	0.86		
I followed (team name) because I liked (coach name).	4.25	1.80	0.89		
I was a fan of (team name) because they were coached by (coach name).	4.23	1.86	0.88		
**Attachment to the national team during the 2002 FIFA World Cup**				0.89	0.74
One reason why I was a fan of (team name) was because it increased the status of our community.	5.39	1.41	0.86		
I was a fan of (team name) because it enhanced the community image.	5.39	1.41	0.83		
The reason I was a (team name) fan was because the team provided the nation’s perception of the state of (team name).	5.17	1.52	0.89		
**Attachment to football during the 2002 FIFA World Cup**				0.81	0.59
First and foremost, I considered myself a football fan.	5.72	1.29	0.89		
Football was my favorite sport.	5.80	1.20	0.84		
I was a football fan at all levels (e.g., high school, college, professional).	4.82	1.62	0.52		

*M, mean; SD, standard deviation; λ, standardized factor loadings; CR, composite reliability; AVE, average variance extracted.*

Translation checks for the scales were conducted to minimize discrepancies between the original English version and translated Korean and Japanese versions. The authors first translated the English items into Korean and Japanese. Two Korean and two Japanese bilingual researchers assessed the accuracy of the translated scales. Furthermore, two bilingual researchers of Korean and Japanese examined the differences in meaning between the Korean and Japanese scales. Although a few minor changes were recommended, they stated that there was no major difference in meaning between the two versions of the assigned scales. Based on this, the authors revised several terms.

For data analyses, a hierarchical multiple regression analysis was performed to test the hypotheses by employing four POAs to the 2002 FIFA World Cup as independent variables. Several control variables (e.g., nationality and age) and two dependent variables (i.e., football participation frequency immediately after the 2002 FIFA World Cup and the present frequency of football participation) were included in the regression model. Hierarchical regression analysis effectively determines whether each independent variable explains a statistically significant amount of incremental variance in the dependent variable (Rutter and Gatsonis, 2001; Jensen et al., 2020). Before the regression analysis, a confirmatory factor analysis was performed to check the measurement model. Common method bias, multicollinearity, and test–retest reliability were also confirmed.

## Results

### Measurement Model

A confirmatory factor analysis was performed. The model fit showed acceptable levels for all indices (χ^2^/*df* = 2.851, GFI = 0.932, AGFI = 0.876, CFI = 0.905, RMSEA = 0.048; Hu and Bentler, 1999; Hair et al., 2009), indicating an acceptable model fit to the data. The standardized factor loadings of all items were statistically significant and ranged from 0.52 to 0.92, surpassing the cutoff point of 0.50 (Hair et al., 2009; Table 1). The internal consistency of each variable was measured using composite reliability (CR). The CR values ranged from 0.81 to 0.92, indicating acceptable levels of reliability for the variables according to the recommended 0.60 threshold (Bagozzi and Yi, 1988). Convergent validity was evaluated using the average variance extracted (AVE). The AVE values were greater than the 0.50 standard for convergent validity (Fornell and Larcker, 1981), ranging from 0.59 to 0.80. These variables showed acceptable convergent validity. To examine discriminant validity, the squared correlations between the measured variables were analyzed. The AVE for each variable was greater than the squared correlations (Table 2), supporting discriminant validity (Fornell and Larcker, 1981). Taken together, the measurement model was successfully fitted to the data.

**TABLE 2:** Correlation matrix of the latent variables.

Variables	1	2	3	4	5
1. Inspiration from experience with the 2002 FIFA World Cup	***0.80***	0.01	0.05	0.20	0.19
2. Attachment to the player during the 2002 FIFA World Cup	0.10	***0.72***	0.39	0.11	0.09
3. Attachment to the coach during the 2002 FIFA World Cup	0.22	0.62	***0.77***	0.30	0.05
4. Attachment to the national team during the 2002 FIFA World Cup	0.45	0.34	0.55	***0.74***	0.04
5. Attachment to football during the 2002 FIFA World Cup	0.44	0.30	0.22	0.20	***0.59***

*The diagonal (in bold and italics) shows the average variance extracted value for each variable.*

*The correlations are under the diagonal, and the squared correlations are above the diagonal.*

### Tests of Common Method Bias, Multicollinearity, and Test–Retest Reliability

The common method bias was tested since the data were collected using self-reported questionnaires. In the survey question design stage, a variety of recommended procedural techniques were applied: proximal separation of predictor and criterion variables in the survey, variation of scale end labels, randomizing the order of question items to avoid response sets, and carefully constructing questions adapting previously validated scales and questionnaires kept short to prevent ambiguity (Podsakoff et al., 2012). Along with procedural remedies, principal components factor analysis, according to Harman’s one-factor test, was performed. The results showed that the most significant explained variance before rotation (26.4%) was lower than the recommended 50.0% threshold (Podsakoff et al., 2003). Therefore, it was confirmed that no serious common method bias exists.

Multicollinearity among variables was tested by measuring the variance inflation factor (VIF), square root of the VIF, and tolerance criteria. Every variable showed a VIF that was less than 10.00, the square root of the VIF less than 2.00, ranging from 1.04 to 1.53, and tolerance at more than 0.10 (Table 3), signifying that multicollinearity was not a concern (Kutner et al., 2004). The test–retest reliability between the football participation frequency immediately after the 2002 FIFA World Cup and the present frequency of football participation was assessed. Intraclass correlation coefficient (ICC) values (model: two-way mixed, type: absolute agreement) showed good reliability between the frequencies of football participation [ICC = 0.72; 95% CI (0.52, 0.82); Landis and Koch, 1977], indicating that the test–retest reliability was acceptable.

**TABLE 3:** Variables of the research model and results of multicollinearity test.

Variables	Type	VIF	Tolerance
**Control variables**			
Nationality	Category (0 = Korea, 1 = Japan)	2.28	0.44
Age	Numeric	1.09	0.92
Watched a game of the 2002 FIFA World Cup at the stadium	Category (0 = No, 1 = Yes)	1.13	0.88
Watched a game of the 2002 FIFA World Cup on TV	Category (0 = No, 1 = Yes)	1.13	0.88
Watched a game of the 2002 FIFA World Cup at the public viewing site	Category (0 = No, 1 = Yes)	1.74	0.57
Inspiration from experience with the 2002 FIFA World Cup	Numeric (a 7-point Likert scale)	1.56	0.64
**Independent variables**			
Attachment to the player during the 2002 FIFA World Cup	Numeric (a 7-point Likert scale)	1.87	0.54
Attachment to the coach during the 2002 FIFA World Cup	Numeric (a 7-point Likert scale)	2.35	0.43
Attachment to the national team during the 2002 FIFA World Cup	Numeric (a 7-point Likert scale)	1.82	0.55
Attachment to football during the 2002 FIFA World Cup	Numeric (a 7-point Likert scale)	1.55	0.65
**Dependent variable 1**			
Football participation frequency immediately after the 2002 FIFA World Cup	Numeric (a 7-point Likert scale)	–	–
**Dependent variable 2**			
Present frequency of football participation	Numeric (a 7-point Likert scale)	–	–

### Hypothesis Testing

Hypothesis testing was performed using a two-step hierarchical multiple regression analysis. In the first step, six control variables were included in the regression model: nationality; age; experience watching a game of the 2002 FIFA World Cup at the stadium, on TV, and at the public viewing site; and inspiration from experience of the 2002 FIFA World Cup. Except for age and inspiration, the four variables were coded according to category type. Nationality was coded 0 for South Korea and 1 for Japan; experiences watching a game of the 2002 FIFA World Cup at the stadium, on TV, and at the public viewing site were coded 0 for “no experience” and 1 for “experienced” (Table 3). Age was numeric, and inspiration was rated on a 7-point Likert-type scale. Four POAs to the 2002 FIFA World Cup were added to the second step as predictors. In the regression model that employed the present frequency of football participation as a dependent variable, football participation frequency immediately after the 2002 FIFA World Cup was added to the first step as a control variable.

Hypotheses were tested. The results of the second step regression analysis showed that with regard to the effects on football participation frequency immediately after the 2002 FIFA World Cup (Δ*R*^2^ = 0.11, *p* < 0.001; Table 4), all four POAs to the 2002 FIFA World Cup were statistically significant and positive (attachment to the player: β = 0.13, *p* < 0.01; attachment to the coach: β = 0.09, *p* < 0.05; attachment to the national team: β = 0.20, *p* < 0.001; attachment to football: β = 0.10, *p* < 0.05). Therefore, H1a, H2a, H3a, and H4a are supported.

**TABLE 4:** Results of hierarchical multiple regression analysis regarding football participation frequency immediately after the 2002 FIFA World Cup.

Variables	Step 1		Step 2	
	β	*p*	β	*p*
**Control variables**				
Nationality	−0.17	<0.001	−0.09	0.05
Age	−0.00	0.94	−0.02	0.49
Watched a game of the 2002 FIFA World Cup at the stadium	0.15	<0.001	0.11	<0.001
Watched a game of the 2002 FIFA World Cup on TV	−0.04	0.26	−0.04	0.20
Watched a game of the 2002 FIFA World Cup at the public viewing site	0.06	0.13	0.08	<0.05
Inspiration from experience with the 2002 FIFA World Cup	0.28	<0.001	0.12	<0.001
**Independent variables**				
Attachment to the player during the 2002 FIFA World Cup	–	–	0.13	<0.01
Attachment to the coach during the 2002 FIFA World Cup	–	–	0.09	<0.05
Attachment to the national team during the 2002 FIFA World Cup	–	–	0.20	<0.001
Attachment to football during the 2002 FIFA World Cup	–	–	0.10	<0.05
*R* ^2^	0.18 (*p* < 0.001)		0.29 (*p* < 0.001)	
Δ*R*^2^	0.18 (*p* < 0.001)		0.11 (*p* < 0.001)	
*F*-value	28.34		31.60	

*β, standardized beta coefficient.*

H1b, H2b, H3b, and H4b were tested. With regard to the effects on the present frequency of football participation, the results of the second step regression analysis (Δ*R*^2^ = 0.06, *p* < 0.001; Table 5) showed that only two POAs to the 2002 FIFA World Cup were statistically significant and positive (attachment to the player: β = 0.08, *p* < 0.05; attachment to the coach: β = 0.18, *p* < 0.001). These results support H1b and H2b, but not H3b and H4b.

**TABLE 5:** Results of hierarchical multiple regression analysis regarding the present frequency of football participation.

Variables	Step 1		Step 2	
	β	*p*	β	*p*
**Control variables**				
Nationality	−0.09	<0.05	−0.04	0.26
Age	0.05	0.06	0.04	0.10
Watched a game of the 2002 FIFA World Cup at the stadium	0.08	<0.01	0.06	<0.05
Watched a game of the 2002 FIFA World Cup on TV	−0.02	0.52	−0.03	0.35
Watched a game of the 2002 FIFA World Cup at the public viewing site	0.00	0.94	0.02	0.51
Inspiration from experience with the 2002 FIFA World Cup	0.01	0.68	−0.05	0.16
Football participation frequency immediately after the 2002 FIFA World Cup	0.60	<0.001	0.50	<0.001
**Independent variables**				
Attachment to the player during the 2002 FIFA World Cup	–	–	0.08	<0.05
Attachment to the coach during the 2002 FIFA World Cup	–	–	0.18	<0.001
Attachment to the national team during the 2002 FIFA World Cup	–	–	0.04	0.30
Attachment to football during the 2002 FIFA World Cup	–	–	0.06	0.06
*R* ^2^	0.43 (*p* < 0.001)		0.48 (*p* < 0.001)	
Δ*R*^2^	0.43 (*p* < 0.001)		0.06 (*p* < 0.001)	
*F*-value	84.15		67.31	

*β, standardized beta coefficient.*

## Discussion and Implications

This study aimed to examine which POA to the 2002 FIFA World Cup promoted football participation frequency immediately after the event and the present frequency of football participation in host countries. An online questionnaire survey was conducted in the host countries. The results of hierarchical regression analyses indicated that those who had a higher attachment to each point (players, coaches, national teams, and football) during the 2002 FIFA World Cup reported a higher frequency of football participation immediately after the event. However, only two higher POAs—the player and the coach—induced a higher frequency of present football participation. The theoretical and practical implications of this study are discussed below.

### Theoretical Implications

This study extends the literature in three ways. First, the results demonstrate the role of the POA in the 2002 FIFA World Cup in enhancing the frequency of mass football participation in the hosting regions after the event. These results can contribute to previous arguments on the necessity for further studies to identify the conditions under which the trickle-down effect of mega-sporting events on mass sport participation has remained (Veal et al., 2012; Hahm et al., 2020; Potwarka and Wicker, 2021). Despite positive evidence of the trickle-down effect in the sporting context (Veal et al., 2012; Ramchandani et al., 2015; Chen and Henry, 2016; Potwarka and Leatherdale, 2016; Kokolakakis et al., 2019), doubts regarding its validity have also been raised in some studies (Charlton, 2010; Sousa-Mast et al., 2013; Taks et al., 2018). The findings of this study can further justify the trickle-down effect on mass sport participation in mega-sporting events, as in the case of the 2002 FIFA World Cup. Particularly, consistent with Weed et al.’s (2015) suggestion on realizing specific designs for boosting mass sport participation legacy through mega-sporting events, our findings are expected to provide specific ways to design the trickle-down effect of mega-sporting events by highlighting the relationships between POA, a well-developed concept in the field of sport management, during the 2002 FIFA World Cup and mass football participation.

Second, the results suggest that the trickle-down effect of the 2002 FIFA World Cup could be better understood by measuring it at different time points. Regarding the impact on increasing football participation frequency immediately after the 2002 FIFA World Cup, all four POAs to the event were significantly positive. Such results were as hypothesized, and can be attributed to the expectation that participants who had a high attachment to the 2002 FIFA World Cup during the event were affected by the trickle-down effect of the 2002 FIFA World Cup more than those with a low attachment. Therefore, the findings highlight the importance of a high POA in generating a trickle-down effect immediately after mega-sporting events, including the 2002 FIFA World Cup, in terms of mass sport participation. In contrast, only two POAs to the 2002 FIFA World Cup, players and coaches, were positively significant in increasing the present frequency of football participation. These results indicate that a high level of attachment to the national team and football increased the frequency of football participation immediately after the event; however, such effects were not sustained over time. With regard to the non-significance of attachment to the national team, the authors can expect it based on previous research arguing that for goals to produce the lasting trickle-down effect of mega-sporting events, an effective method is to recall individual memories by focusing on sources more relevant to the overall event experience or personal identity, rather than a peripheral experience, such as national identity (Hahm et al., 2020). Although a high attachment to the national team was found to be critical to the short-term effect on football frequency, it might be a less target-oriented POA than others (i.e., the player and coach) since the national team exists only during national contests. Considering such characteristics, the impact of high attachment to the national team might be difficult to last long term, perhaps being limited to the football frequency 17 years after the 2002 FIFA World Cup. Meanwhile, as for the non-significance of the attachment to football, it can be expected that those who were strongly attached to football at that time already had a higher frequency of football participation before the 2002 FIFA World Cup. For this assumption, its short-term effect on football participation was comparatively low (β = 0.10), possibly resulting in a lack of long-term effects. Consequently, the findings of this research first demonstrate different viewpoints on the relationships between the POA to the 2002 FIFA World Cup and the football participation legacy.

Third, several control variables added more insight into the trickle-down effect of the 2002 FIFA World Cup. Nationality and the experience of watching a game of the 2002 FIFA World Cup at the stadium were positively significant in both the short- and long-term effects of increasing football frequency. As for the more positive effect on South Korea than on Japan (β = −0.17 in the first step of Table 4; β = −0.09 in the first step of Table 5), the final results achieved by the national teams were expected to be significant. Both countries achieved their best ever World Cup results, but the former made it to the semi-finals, while the latter only made it to round 16. Such divergent results might have led to a more positive impact on South Korea. Regarding the significant and positive effect of the experience of watching a game of the 2002 FIFA World Cup at the stadium (β = 0.15 in the first step of Table 4; β = 0.08 in the first step of Table 5), since the experience at the stadium can be more vivid than on TV and at the public viewing site, its impact might have generated a significant trickle-down effect. Meanwhile, different results were indicated: the positive effect of inspiration from experience with the 2002 FIFA World Cup on football participation frequency immediately after the 2002 FIFA World Cup (β = 0.28 in the first step of Table 4), and the positive effect of football participation frequency immediately after the 2002 FIFA World Cup on the present frequency of football participation (β = 0.60 in the first step of Table 5). The former effect represents how important not only the experience but also the inspiration from such an experience is to promote the short-term impact, whereas the latter effect represents the strong link between the short-term and long-term impacts of mass football participation.

### Practical Implications

The current findings can be advantageous for hosting cities and organizers, one of which is to stimulate mass sports participation through mega-sporting events. They can consider spectators’ POA for the event when designing a mass sport participation legacy. Many organizers often focus on activating individuals’ identities with the national team because their attachment to the national team becomes extremely high during the event. Through such efforts, they could achieve short-term effects, including sport participation, which might last a lifetime. However, given the non-significant effect of attachment to the national team on present football participation in this research, it may no longer be effective to emphasize national identity during and after the event. Instead, drawing on the positive effects of attachment to players and coaches, more target-oriented POAs significantly encouraged the continuing effect on sport participation legacy. Therefore, activities to boost attachment toward others as well as the national team or promote interest in the national team to be connected to the interests of players or coaches are needed for those who want to design a lasting sport participation legacy. Alternately, based on the most significant impact of the increased football participation frequency immediately after the 2002 FIFA World Cup on the present frequency of football participation, efforts to link the short-term and long-term effects can be recommended. As mentioned above, this study suggests that the high interest in the national team during the event can last as other interests, such as players, after the event. The findings imply the likelihood that even though individuals had a comparatively low attachment to the player or coach, their present football participation frequency could continue because of the significantly increased football participation frequency immediately after the event. Therefore, organizers are recommended to conceive and initiate efforts toward such individuals.

## Limitations and Future Research

Although this study can help to better understand the trickle-down effect of the 2002 FIFA World Cup on mass football participation in host countries, it also has several limitations. Since the current study conducted surveys based on the memories that participants experienced during the 2002 FIFA World Cup, the results have not been fully identified on a longitudinal basis. Furthermore, even if the participants provided correct answers to the recall questions of the 2002 FIFA World Cup, the authors must acknowledge the possibility that their memories might be biased. There may be other variables that could interfere with the relationship between events and participation. Particularly, given a low level of *R*^2^ (0.18) regarding the effect on enhancing football participation frequency immediately after the 2002 FIFA World Cup, more variables (e.g., socio-cultural backgrounds) can be added to the model as control variables. Other POA dimensions (e.g., attachment to the level of sports) should be considered in future research based on its research context because of the importance of contemplating the research context when establishing POA dimensions. This study was conducted using the context of the 2002 FIFA World Cup only, which may reduce the generalizability of the strong relationship between POA, trickle-down effect, and sport participation. Further studies using other contexts are required to improve the generalizability of the results. Finally, the participants of this study were only males, which may limit the generalizability of the results. Considering the growing popularity of football among women in both countries, this offers an immense scope for future studies to examine its relationships with cross-gender analyses.

## Data Availability Statement

The raw data supporting the conclusions of this article will be made available by the authors, without undue reservation.

## Ethics Statement

Ethical review and approval was not required for the study on human participants in accordance with the local legislation and institutional requirements. The patients/participants provided their written informed consent to participate in this study.

## Author Contributions

TK conceived and designed the research, analyzed the data, and drafted the manuscript. JH collected the data and reviewed and supervised the manuscript. HM administered the study and edited the manuscript. All authors have contributed to the manuscript and approved the submitted version.

## Conflict of Interest

The authors declare that the research was conducted in the absence of any commercial or financial relationships that could be construed as a potential conflict of interest.

## Publisher’s Note

All claims expressed in this article are solely those of the authors and do not necessarily represent those of their affiliated organizations, or those of the publisher, the editors and the reviewers. Any product that may be evaluated in this article, or claim that may be made by its manufacturer, is not guaranteed or endorsed by the publisher.
